# Stable coevolutionary regimes for genetic parasites and their hosts: you must differ to coevolve

**DOI:** 10.1186/s13062-018-0230-9

**Published:** 2018-12-14

**Authors:** Faina Berezovskaya, Georgy P. Karev, Mikhail I. Katsnelson, Yuri I. Wolf, Eugene V. Koonin

**Affiliations:** 1 Department of Mathematics, Howard University, Washington, DC 20059 USA; 2 National Center for Biotechnology Information, National Library of Medicine, National Institutes of Health, Bethesda, MD 20894 USA; 3 Institute for Molecules and Materials, Radboud University, 6525AJ Nijmegen, Netherlands

## Abstract

**Background:**

Genetic parasites are ubiquitous satellites of cellular life forms most of which host a variety of mobile genetic elements including transposons, plasmids and viruses. Theoretical considerations and computer simulations suggest that emergence of genetic parasites is intrinsic to evolving replicator systems.

**Results:**

Using methods of bifurcation analysis, we investigated the stability of simple models of replicator-parasite coevolution in a well-mixed environment. We first analyze what appears to be the simplest imaginable system of this type, one in which the parasite evolves during the replication of the host genome through a minimal mutation that renders the genome of the emerging parasite incapable of producing the replicase but able to recognize and recruit it for its own replication. This model has only trivial or “semi-trivial”, parasite-free equilibria: an inefficient parasite is outcompeted by the host and dies off, whereas an efficient one pushes the host out of existence, leading to the collapse of the entire system. We show that stable host-parasite coevolution (a non-trivial equilibrium) is possible in a modified model where the parasite is qualitatively distinct from the host replicator in that the replication of the parasite depends solely on the availability of the host but not on the carrying capacity of the environment.

**Conclusions:**

We analytically determine the conditions for stable coevolution of genetic parasites and their hosts coevolution in simple mathematical models. It is shown that the evolutionary dynamics of a parasite that initially evolves from the host through the loss of the ability to replicate autonomously must substantially differ from that of the host, for a stable host-parasite coevolution regime to be established.

**Electronic supplementary material:**

The online version of this article (10.1186/s13062-018-0230-9) contains supplementary material, which is available to authorized users.

## Reviewers

This manuscript was reviewed by Olivier Tenaillon, Sandor Pongor, and Alex Best. For the complete reports, go to the Reviewers' Reports section.

## Background

Genetic parasites are ubiquitous among cellular organisms [1]. In fact, most organisms host a variety of mobile genetic elements (MGE) that differ in their reproduction strategies and the mode of parasite-host interaction, including transposons, plasmids and viruses [2]. The abundance of the MGE in the biosphere is enormous. Viruses are by far the most common biological entities on earth [3–6], genes of MGE, such as those encoding transposases, are among the most abundant ones in diverse environments [7–9], and the genomes of many multicellular organisms consist of up to 50% integrated MGE, in the case of mammals, or even up to 90% in the case of plants [10, 11].

The entire history of life can be properly depicted only as the perennial coevolution of cellular organisms with genetic parasites that includes both the proverbial arms race and various forms of cooperation [1, 12, 13]. Moreover, multiple lines of evidence point to a major role of genetic parasites in the evolution of biological complexity, in general, and in major transitions in evolution, in particular [14, 15].

The ubiquity and the enormous abundance of the MGE in the biosphere imply that genetic parasitism is an intrinsic feature of life. Indeed, parasites invariably emerge in computer simulations of the evolution of simple replicator systems which, in well-mixed models, typically leads to the collapse of the entire system [16–20]. This outcome can be avoided by incorporating compartmentalization into the model [18–20]. Further, mathematical models of the evolution of genomes with integrated MGE, combined with probabilistic reconstruction of the evolution of bacterial and archaeal genomes, suggest that horizontal gene transfer at rates that are required to stave off the mutational meltdown of microbial populations (Muller’s ratchet) prevents elimination of genetic parasites [21].

Cellular organisms and genetic parasites have been considered the two “empires” of life that fundamentally differ with regard to the capability of autonomous reproduction [22, 23]. The MGE fully depend on the host for energy production and the biosynthetic processes, in particular, translation (notwithstanding the fact that many large viruses encode components of the respective functional systems that modify and modulate the respective host functions [24, 25]).

In our previous theoretical analysis of the evolution of genetic parasites in simple replicator systems [26] which was, to a large extent, inspired by the seminal early experiments of Spiegelman and colleagues on reductive evolution of bacteriophages genomes in vitro [27–30], we present a semi-formal argument that the parasite-free state of a replicator is inherently evolutionarily unstable. Here, we explore these and derivative models analytically and show that, in order for a replicator and a parasite to stably coevolve, the parasite must substantially differ from the host in its reproduction strategy.

## Results and discussion

### Genetic parasite as a degraded variant of the replicator

A simple conceptual model of the emergence of a genetic parasite from within a self-replicating system [26] implies (initially infinitesimal) degradation of the replicase-encoding signal while the replicase-recognition signal is retained. The dynamics of such a system is described by the following pair of ordinary differential equations [26]:

1 $$ \frac{dR}{dt}=\frac{1}{\left(1+\alpha e\right)}{R}^2\left(1-\frac{R+\frac{P}{q}}{K}\right)-{e}_RR $$

$$ \frac{dP}{dt}=\frac{q}{\left(1+e\right)} RP\left(1-\frac{R+\frac{P}{q}}{K}\right)-{e}_PP $$

(see Table 1 for the model parameters). The model is based on the following assumptions:

- The kinetics of replication depends on the interaction of the replicase and the template (either the host replicator or a parasite), so that the replication rate is proportional to the product of the respective population sizes (*R*^2^ for the replicator and *RP* for a parasite that lacks independent replication ability)
- The decay rates are constant (*e*_*r*_ and *e*_*P*_) for replicator and parasite respectively
- The parasite template replicates faster than that of the replicator by the factor *q* ≥ 1, which, as the first approximation, could interpreted as the “economy” factor (the simplest interpretation is parasite being *q* times smaller than the replicator, so it’s replication is faster by the factor of *q*)
- Both populations are environmentally limited by the same resources; the parasites consume *q* times less resources per individual compared to the host replicator; the environment carrying capacity *K* determines the point where replication becomes resource-limited
- The replicators possess a (costly) defense mechanism with efficiency *e* ≥ 0 that is capable to suppress the parasite replication by a factor of 1 + *e* at the cost to the replicators replication rate of 1 + *αe* (where *α* ≥ 0 is the defense mechanism cost factor)

**Table 1:** Parameters of the replicator-parasite dynamics model

Name	Description
*e*_*R*_	Replicator intrinsic decay rate
*e*_*P*_	Parasite intrinsic decay rate
*e*	Defense system efficiency
*α*	Cost of defense system
*q*	Parasite advantage factor
*K*	Carrying capacity of the environment

As noticed previously [26], this system has equilibria ($R=\Big(K\pm \sqrt{K\left(K-4{e}_R\right)}/2$, *P* = 0) as *e* = 0. The point $R=\Big(K-\sqrt{K\left(K-4{e}_R\right)}/2$, *P* = 0) is always unstable, that is, introduction of the parasite into a replicator system without any defense near this equilibrium leads to eventual collapse of the entire system. The same happens at the equilibria $R=\Big(K+\sqrt{K\left(K-4{e}_R\right)}/2$, *P* = 0) if *q* > *e*_*p*_/*e*_*R*_. If *q* < *e*_*p*_/*e*_*R*_, then this equilibrium is stable, and introduction of the parasite into the system leads to runaway parasite proliferation.

Here, we aim to identify all stationary states of model (1) and to analyze their stability, to study the qualitative behavior of this model and to suggest some modifications that make possible the stable host-parasite co-evolution.

**Fig. 1:**
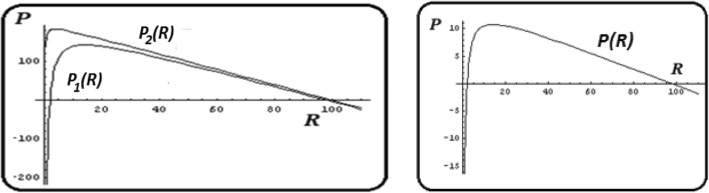
Non-trivial null-clines *P*_1_(*R*), *P*_2_(*R*)for model (1). See eq.(2). Left panel: for *q*(1 + *αe*)*e*_*R*_ ≠ (1 + *e*)*e*_*P*_), the isoclines *P*_1_(*R*), *P*_2_(*R*) do not intersect for positive *P*, *R*. Right panel: *P*_1_(*R*)≡ *P*_2_(*R*) for *q*(1 + *αe*)*e*_*R*_ = (1 + *e*)*e*_*P*_), so system (1) has a line of non-isolated equilibria

All possible equilibria of the model can be found from the system of equations $\frac{dP}{dt}=0,\frac{dR}{dt}=0$. These equations determine the following pair of (non-zero) isoclines (see Additional file 1: Mathematical Appendix 1 for details):

2 $$ {P}_1(R)=\frac{q\left(R\left(K-R\right)-{e}_RK\left(1+\alpha e\right)\right)}{R} $$

$$ {P}_2(R)=\frac{q\ R\left(K-R\right)-{e}_PK\left(1+e\right)}{R} $$

the intersection of which (*P*_1_(*R*) = *P*_2_(*R*) at *R* > 0, *P* > 0) indicates a non-trivial equilibrium. It should be immediately apparent, however, that both equations have the same form and differ only by the values of the coefficients *q*(1 + *αe*)*e*_*R*_ and (1 + *e*)*e*_*P*_, respectively (see Fig. 1, left panel, for the characteristic shapes of these isoclines when *q*(1 + *αe*)*e*_*R*_ ≠ (1 + *e*)*e*_*P*_). Therefore, the curves do not intersect in any point (*R* > 0, *P* > 0); the only case where such equilibria exist is $q=\frac{\left(1+e\right){e}_P}{\left(1+\alpha e\right){e}_R}$; in this case *P*_1_(*R*) = *P*_2_(*R*) over the whole range of *R* and the system has a line of non-isolated equilibria (see Fig. 1, right panel).

More formally, model (1) always has the trivial equilibrium *O*(*R* = 0, *P* = 0) (both populations collapse). In addition to that, the model might have up to two parasite-free, “semi-trivial” equilibria

3 $$ {O}_1\left(R=\frac{K-\sqrt{K\Big(K-4\left(1+\left(1+\alpha e\right){e}_R\right)}}{2},P=0\right) $$

$$ {O}_2\left(R=\frac{K+\sqrt{K\Big(K-4\left(1+\left(1+\alpha e\right){e}_R\right)}}{2},P=0\right) $$

the existence of which depends on the value of the following expression:

4 $$ E=K-4\left(1+\left(1+\alpha e\right){e}_R\right) $$

If *E* > 0, both *O*_1_ and *O*_2_ exist; if *E* = 0, *O*_1_ = *O*_2_ = *O*_12_(*R* = *K*/2, *P* = 0); if *E* < 0, the model has only the trivial equilibrium *O*.

**Fig. 2:**
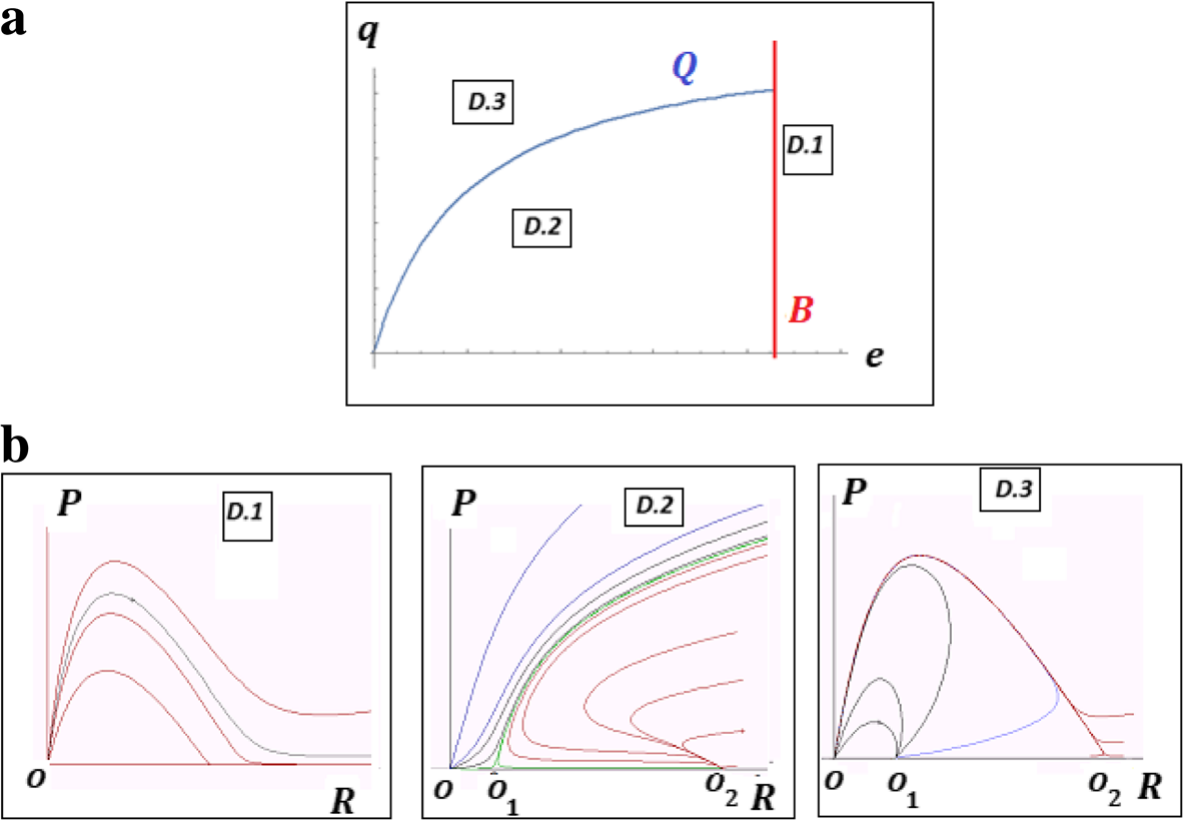
Characteristics of model (1). **a** Parameter-phase portrait. ***D*1**, ***D*2** and ***D*3** are the domains with qualitatively different model behaviors. The boundaries are: $\boldsymbol{Q}:q=\frac{\left(1+e\right){e}_P}{\left(1+\alpha e\right){e}_R}$(blue), $\boldsymbol{B}:e=\frac{K-4{e}_R}{4\alpha {e}_R}$ (red). **b** Examples of phase portraits of model (1) with a stable trivial equilibrium *O* (all domains); stable semi-trivial equilibrium *O*_2_ and unstable semi-trivial equilibrium *O*_1_ (domain ***D*2**); unstable semi-trivial equilibria *O*_1_, *O*_2_ (domain ***D*3**)

**Fig. 3:**
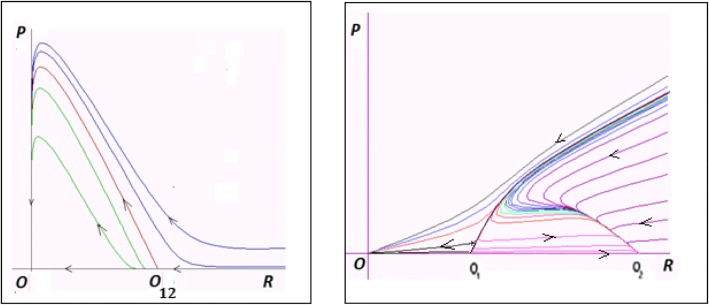
Phase portraits of the model (1) on the boundaries *B* (left panel) and *Q* (right panel) of the phase- parametric portrait

Stability analysis shows that the trivial equilibrium *O* is a stable node at all values of the model parameters. When both *O*_1_ and *O*_2_ exist (*E* > 0), if $q<\frac{\left(1+e\right){e}_P}{\left(1+\alpha e\right){e}_R}$, *O*_1_ is a saddle and *O*_2_ is a stable node and if $q>\frac{\left(1+e\right){e}_P}{\left(1+\alpha e\right){e}_R}$, *O*_1_ is an unstable node and *O*_2_ is a saddle. If *K* = 4(1 + *e*)/*q*, the only semi-trivial equilibrium *O*_12_ is a saddle-node fixed point (Fig. 2b; see Additional file 1: Mathematical Appendix 1 for details). Let us consider *q* and *e* as parameters of model (1) whereas *K*, *α*, *e*_*R*_, *e*_*P*_ are arbitrary values of fixed coefficients. The parametric portrait of the system can be divided into 3 domains, ***D1***, ***D2*** and ***D3*** with qualitatively different behaviors of the model (Fig. 2a); these domains are defined by Theorem 1 (Fig. 3 and Additional file 1: Mathematical Appendix 1). Asymptotically, as *t* → ∞, there exist two qualitatively different types of behavior of the system, namely:

1. If the parameters *q*, *e* belong to the domains ***D1*** or ***D3*** then, for any initial values (*R*, *P*), the system collapses (*R* → 0, *P* → 0);
2. If the parameters *q*, *e* belong to the domain ***D2***, then, there exist domains of initial values in which the system tends to the parasite-free equilibrium *O*_2_ whereas, at other initial values, it tends to the trivial equilibrium *O* (collapse).

Therefore, the original model [26] has no non-trivial (*R* > 0, *P* > 0) equilibria whereby the replicator and the parasite could coexist and coevolve. A stable parasite-free equilibrium can exist only if $q<\frac{\left(1+e\right){e}_P}{\left(1+\alpha e\right){e}_R}$ and necessarily exists if, additionally, *ae* < (*K* − 4*e*_*R*_)/(4*e*_*R*_)), that is, if the reproductive advantage of the parasite is low and the replicator possesses defense mechanisms that are efficient enough and not too costly to overcome the intrinsic advantage of the parasite.

The implications of these findings are the following: emergence of a genetic parasite as a (slightly) degraded copy (e.g. a variant of an RNA genome that contains a deletion and thus cannot produce an active replicase) of the “naïve” (defenseless) replicator (i.e. when eqs. (1) apply and *e* = 0) leads to the collapse of the system towards the trivial equilibrium (*R* = 0, *P* = 0). If the replicator already has a sufficiently advanced and not excessively costly defense mechanism (or is able to evolve it before the population collapse), the system could be stable in the parasite-free state as long as the defense mechanism persists. If the defense degrades over time, which is likely to be the case because most if not all defense mechanisms incur a non-zero fitness cost [31–34], the system will become vulnerable again. Therefore, such a system is inherently unstable. If the early history of (pre-)life included a primitive replicator stage, as the RNA World concept implies [17, 35, 36], it would be vulnerable to parasite-driven collapse, could not have persisted for a long time and necessarily would evolve into a different mode of replicator-parasite relationships that is not subject to the limitations imposed by eq. (1).

### Genetic parasite with an additional interaction with the replicator

Let us consider the model that differs from the model (1) by an additional effect of the replicator-parasite interaction on the replicator dynamics:

5 $$ \frac{dR}{dt}=\frac{1}{\left(1+\alpha e\right)}{R}^2\left(1-\frac{R+\frac{P}{q}}{K}\right)- bRP-{e}_RR $$

$$ \frac{dP}{dt}=\frac{q}{\left(1+e\right)} RP\left(1-\frac{R+\frac{P}{q}}{K}\right)-{e}_PP $$

Such an effect could be interpreted as an extra penalty on the replication rate:

$$ \left[R\frac{1}{\left(1+\alpha e\right)}\left(1-\frac{R+\frac{P}{q}}{K}\right)- bP\right]R $$

and/or as extra replicator degradation rate:

$$ \left[{e}_R+ bP\right]R $$

due to the replicator-parasite interaction.

The trivial equilibrium *O* (*R* = 0, *P* = 0), and the semi-trivial equilibria *O*_1_ and *O*_2_ (*R* > 0, *P* = 0), identified for the model (1) still exist in the system (5) under the same conditions. In addition, however, two new equilibria also can exist in the system, namely, *A*_1_(*R*^+^, *P*^∗^) and *A*_2_(*R*^−^, *P*^∗^) where

$$ {R}^{+,-}=\frac{q\left(1+\alpha e\right){e}_R-\left(1+e\right){e}_P+ bK{q}^2\left(1+\alpha e\right)\pm \sqrt{{\left(\left(1+e\right){e}_P-\left(1+\alpha e\right)q\left({e}_R+ bK q\right)\right)}^2-4\left(1+e\right){b}^2{\left(1+\alpha e\right)}^2{e}_PK{q}^3}}{2b\left(1+\alpha e\right){q}^2} $$

$$ {P}^{\ast }=\frac{\left(1+e\right){e}_P-q\left(1+\alpha e\right){e}_R}{bq\left(1+\alpha e\right)} $$

These equilibria are positive (i.e. imply *P* > 0) only if $q<\frac{\left(1+e\right){e}_P}{\left(1+\alpha e\right){e}_R}$; if $q=\frac{\left(1+e\right){e}_P}{\left(1+\alpha e\right){e}_R}$, the equilibrium points *A*_1_ and *A*_2_ merge with the points *O*_1_ and *O*_2_ of eq. (3) and become negative if $q>\frac{\left(1+e\right){e}_P}{\left(1+\alpha e\right){e}_R}.$

**Fig. 4:**
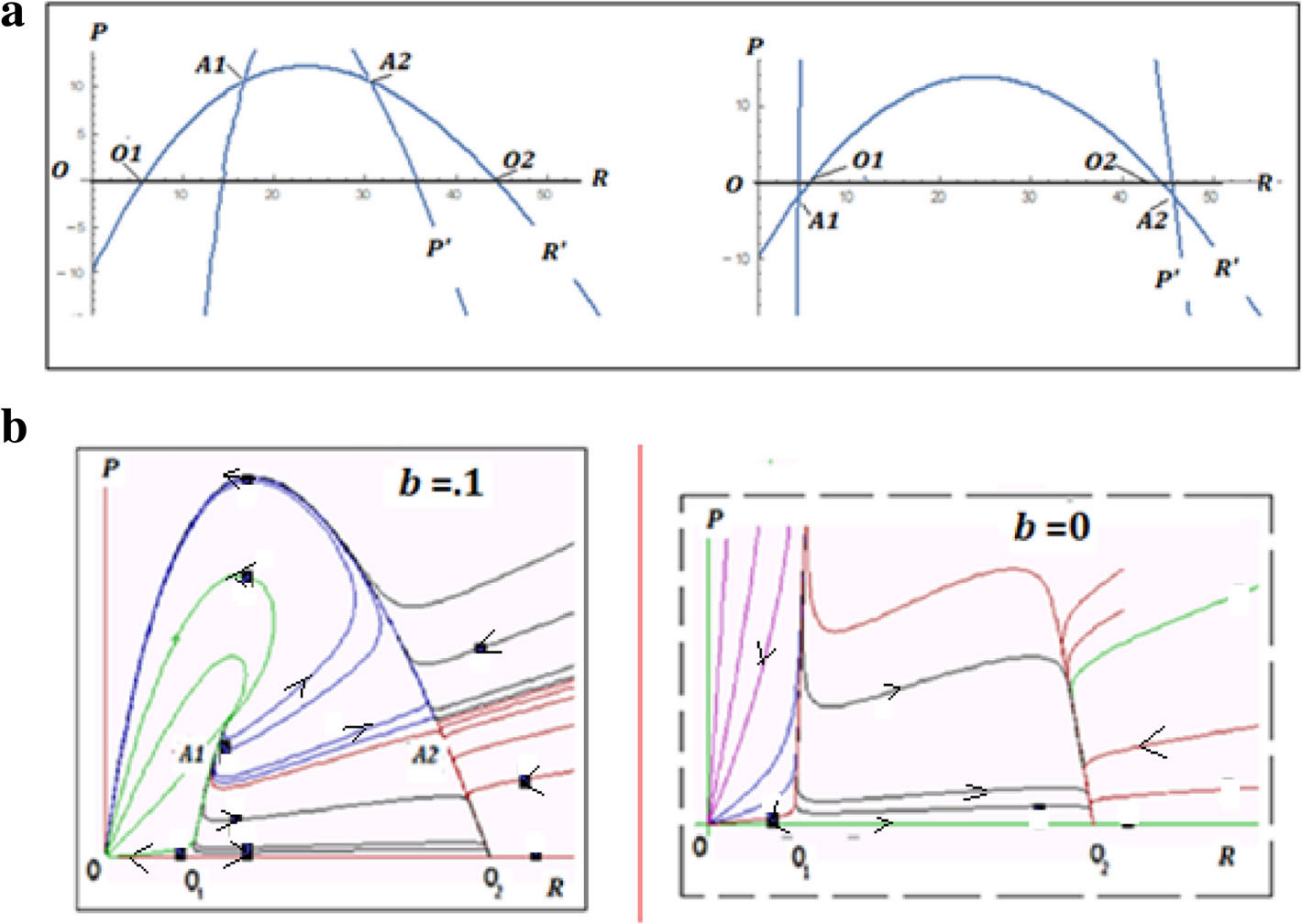
Characteristics of model (5). **a** Null - isoclines and equilibria. Left panel: positive non-trivial equilibria *A*_1_, *A*_2_ as *q* = 4; right panel: no positive non-trivial equilibria as *q* = 10. **b** Phase portraits. Left panel: phase portrait of model (5) as *b* > 0 with unstable non-trivial equilibria *A*_1_ and *A*_2_. Right panel: phase portrait of model (5) as *b* = 0 is similar to the phase portrait of model (1), no non-trivial equilibria

Stability analysis, however, shows that, when these equilibria belong to the first quadrant, both are unstable: *A*_1_ is an *unstable node* and *A*_2_ is a *saddle* (Fig. 4a, b; see Additional file 2: Mathematical Appendix 2 for details). Therefore, although the inclusion of this extra interaction, corresponding to a more aggressive parasite compared to model (1), into the model does lead to non-trivial equilibria (i.e. replicator-parasite coexistence), they are unlikely to persist for on the evolutionarily relevant time scale.

### A highly derived genetic parasite

Analysis of the model (1) shows that the equilibrium between the parasite and the replicator is unstable and largely hinges on the relative growth advantage of the parasite *q* and the efficiency of the host replicator’s defense mechanisms $\frac{\left(1+e\right)}{\left(1+\alpha e\right)}$. A parasite that has a greater advantage over the host replicator than the defense mechanisms can handle overwhelms the system and drives it to collapse, whereas a less efficient parasite is eliminated by the host. Simply shrinking the parasite genome and hence increasing its replicative advantage (*q* ≫ 1), while concomitantly reducing the impact on the replicator dynamics according to the expression $1-\frac{R+\frac{P}{q}}{K}$, does not represent a viable path for the parasite towards the stable coexistence because the increase in the parasite replication efficiency overwhelms the attenuation of its deleterious effect. Moreover, as the analysis of the model (5) indicates, another intuitively plausible path to coexistence, through additional suppression of the replicator (allowing the parasite to escape elimination in the presence of the efficient defense mechanism, for example, by evolving antidefense mechanisms) does not work either. Although non-trivial equilibria can exist in this model, they are unstable.

More generally, the equilibria appear in the system as intersections of the isoclines (see eq. (2)). Thus, to ensure stable coexistence of the host and the parasite, the equations of the replicator and parasite dynamics should substantially differ from each other unlike those in model (1) (see Additional file 1: Mathematical Appendix 1). From the biological perspective, this means that the parasite cannot be, simply, a slightly modified variant of the replicator. Rather, the effects of the replicator and the parasite on each other’s replication should be substantially asymmetric and/or their interactions with the environment should be substantially different. One such possible modification is represented in the following model:

6 $$ \frac{dR}{dt}=\frac{1}{\left(1+\alpha e\right)}{R}^2\left(1-\frac{R+\frac{P}{q}}{K}\right)-{e}_RR $$

$$ \frac{dP}{dt}=\frac{q}{\left(1+e\right)} RP-{e}_PP $$

In the model (6), the parasite dynamics does not depend on the carrying capacity of the environment, whereas the consumption of the resources by the parasite continues to be a factor in the replicator dynamics ($1-\frac{R+\frac{P}{q}}{K}$). A biological model for this behavior is a highly specialized parasite that obtains the necessary resources from the host (effectively, for free) rather than directly from the environment. If the parasite is individually small enough compared to the host, as is the case for many viruses and transposons, at least those that parasitize on eukaryotes, its growth is not limited by the external environmental resources. The mere availability of the host ensures that the resources are sufficiently abundant and is the only limiting factor for the parasite reproduction.

**Fig. 5:**
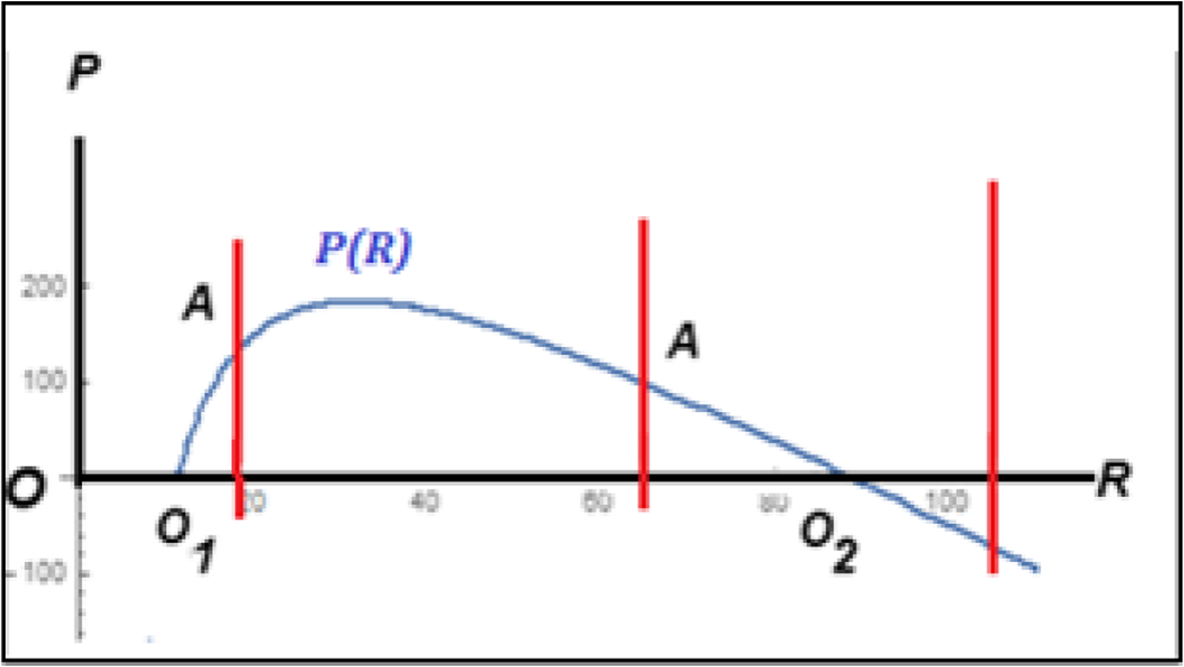
Null isoclines (eq. (7) for model (6)

Analysis of the model equilibria (see Additional file 3: Matematical Appendix 3 for details) defines the following family of null isoclines for *P* and *R*:

$$ {P}_1=0 $$

7 $$ {P}_2(R)=\frac{q\ R\left(K-R\right)-{e}_PK\left(1+e\right)}{R} $$

$$ {R}_1=0 $$

$$ {R}_2=\frac{\left(1+e\right){\mathrm{e}}_P}{q} $$

(Fig. 5). The trivial equilibrium *O* (*R* = 0, *P* = 0), and the semi-trivial equilibria *O*_1_ and *O*_2_ (*R* > 0, *P* = 0), found in model (1) and defined by eq. (3), also exist in model (6) subject to the conditions defined by eq. (4). In addition, an equilibrium

A$$ \left({R}_A=\frac{\left(1+e\right){\mathrm{e}}_P}{q},{P}_A=\frac{Kq\left(1+e\right){\mathrm{e}}_P-{\left(1+e\right)}^2{{\mathrm{e}}_P}^2-\left(1+\upalpha e\right){\mathrm{e}}_RK{q}^2}{\left(1+e\right){\mathrm{e}}_P}\right) $$ (8)

also exists. Under the condition $q=\frac{\left(1+e\right){\mathrm{e}}_P\left(K\pm \sqrt{K\left(K-4\left(1+\upalpha e\right){\mathrm{e}}_R\right)}\right)}{2\left(1+\upalpha e\right){\mathrm{e}}_RK}$, the equilibrium *A* is semi-trivial (*R* > 0, *P* = 0) and coincides with either *O*_1_ or *O*_2_. For the values of *q* defined by

$$ \frac{\left(1+e\right){\mathrm{e}}_P\left(K-\sqrt{K\left(K-4\left(1+\upalpha e\right){\mathrm{e}}_R\right)}\right)}{2\left(1+\upalpha e\right){\mathrm{e}}_RK}<q<\frac{\left(1+e\right){\mathrm{e}}_P\left(K+\sqrt{K\left(K-4\left(1+\upalpha e\right){\mathrm{e}}_R\right)}\right)}{2\left(1+\upalpha e\right){\mathrm{e}}_RK} $$

the equilibrium *A* is non-trivial (*R* > 0, *P* > 0).

**Fig. 6:**
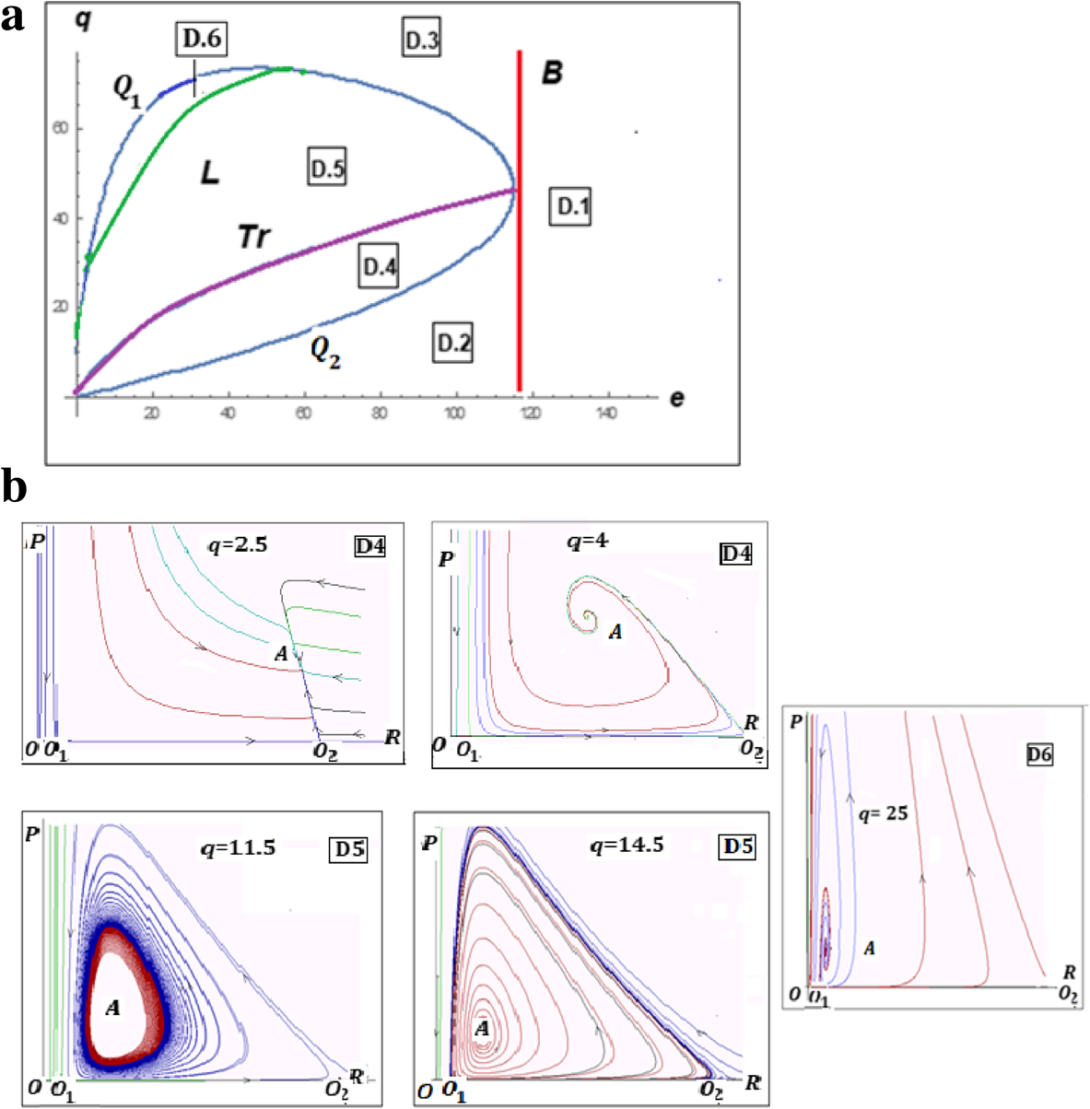
Characteristics of model (6). **a** Phase-parametric portrait of model (6). ***D*1** ***− D*6** are the domains with qualitatively different model behaviors. **b** Examples of phase portraits of model (6) with a stable non-trivial equilibrium *A* (domain ***D*4**) and stable oscillations (domain ***D*5**); no non-trivial stable regimes in domain ***D*6**

Analysis of the model (6) (see Additional file 3: Mathematical Appendix 3 for details) allows us to construct the parameter-phase portrait of the model (Fig. 6a, b). Let us consider *q* and *e* as parameters of the model (6) whereas *K*, *α*, *e*_*R*_, *e*_*P*_ are arbitrary values of fixed coefficients. The portrait contains domains ***D*1**, ***D*2** and ***D*3** similar to those in model (1) (Fig. 2), and 3 additional domains, ***D*4**, ***D*5** and ***D*6**.

**Fig. 7:**
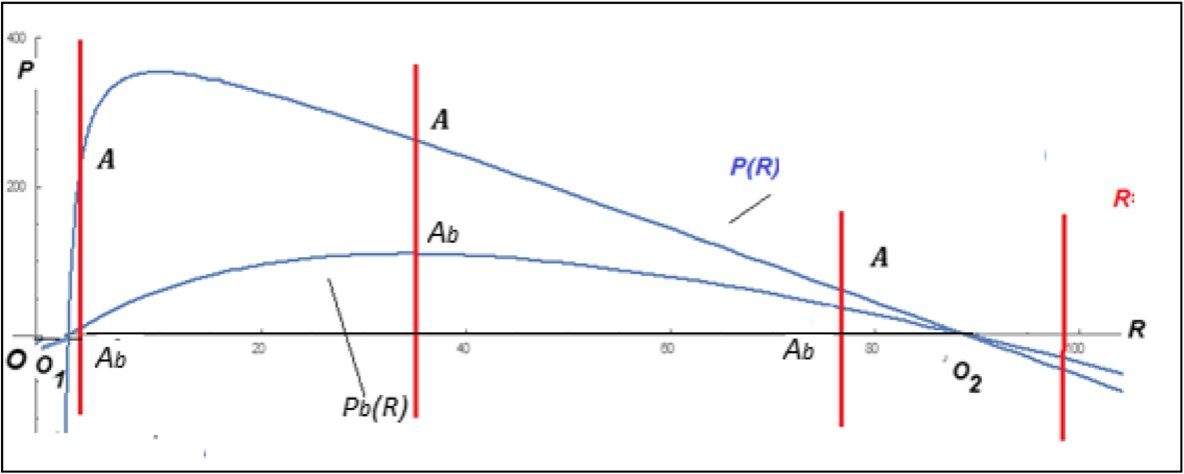
Null isoclines for Volterra-type models. The isocline *P*(*R*) corresponds to the model (A3.1) which is the same as model (6); the isocline *P*_*b*_(*R*) corresponds to the model (A4.1) with *b > 0*

The boundaries between the parametric domains of model (6) are determined by the curves ***B,Q***_**1**_***,Q***_**2**_***,Tr,L*,** described in Additional file 3: Mathematical Appendix 3.

The domains have the following properties:

- ***D*1** (*e* > ***B***): only the trivial equilibrium *O* exists and is stable (in ***D*1**, the effective cost of the defense mechanisms *αe* is so high that the replicator population collapses even in the absence of the parasite);
- ***D*2** (*e* < ***B***, *q* < ***Q***_**1**_): the trivial equilibrium *O* is stable; the semi-trivial equilibrium *O*_1_ is unstable; the semi-trivial equilibrium *O*_2_ is stable; no other equilibria exist (in ***D*2**, the efficiency of the defense mechanisms is sufficient to eliminate the parasite as long as the population of the replicator itself is sustainable);
- ***D*3** (*e* < ***B***, *q* > ***Q***_**2**_): the trivial equilibrium *O* is stable; both semi-trivial equilibria *O*_1_ and *O*_2_ are unstable; no other equilibria exist (in ***D*3**, the parasite overwhelms the defense mechanisms of the replicator and thus collapses the system);
- ***D*4** (*e* < ***B***, ***Q***_**1**_ < *q* < ***Tr***): the trivial equilibrium *O* is stable; both semi-trivial equilibria *O*_1_ and *O*_2_ are unstable; the non-trivial equilibrium *A* is a stable node or focus (in ***D*4**, the parasite and the replicator can coexist at a stable equilibrium, Fig. 6b);
- ***D*5** (*e* < ***B***, ***Tr*** < *q* < min(***L***, ***Q***_**2**_)): the trivial equilibrium *O* is stable; both semi-trivial equilibria *O*_1_ and *O*_2_ are unstable; the non-trivial equilibrium *A* is unstable but is surrounded by a stable limit cycle (in ***D*5**, the parasite and the replicator can coexist in a stable oscillation regime);
- ***D*6** (*e* < ***B***, ***L*** < *q* < ***Q***_**2**_): the trivial equilibrium *O* is stable; both semi-trivial equilibria *O*_1_ and *O*_2_ and the non-trivial equilibrium *A* are unstable (in ***D*6**, the defense mechanisms of the replicator are not sufficient to keep the parasite in check; although other equilibria exist, they are unstable, so that the system would collapse upon a perturbation).

Overall, the system collapses in domains ***D*1**, ***D*3**, and ***D*6**; the replicators and parasites can coexist in domains ***D*4** and ***D*5**; and, the system is bistable in domains ***D*2**, ***D*4** and ***D*5**, that is, its final behavior critically depends on the initial values of *P* and *R*.

Thus, under the model (6), there exists an intermediate regime in which the parasite efficiency is roughly balanced by the efficiency of the replicator defense mechanisms under which the replicator and the parasite can coexist indefinitely in a stable equilibrium or a limit cycle. Unlike in the model (1) that lacks such a regime, the parasite in the model (6) is a highly derived state. In such a state, the parasite replication and survival directly depend only on the host (replicator) availability but not on the environment (the parasite equation in (6) lacks the $1-\frac{R+\frac{P}{q}}{K}$ term). Models with the parasite being closely similar to the replicator in its interaction with the environment (that is, with the parasite replication equation closely resembling that of the replicator), such as the model (1), cannot support stable non-trivial equilibria (compare the families of null isoclines in eqs. (2) and (7)). A variant of the model (6) with an additional effect of the replicator-parasite interaction on the replicator dynamics shows qualitatively the same behavior as model (6) (see Fig. 7 and Additional file 4: Mathematical Appendix 4).

## Concluding remarks

We show here that, in order to produce stable equilibria, models of the coevolution of genetic parasites and their hosts cannot be too simple. The parasite must not be too closely similar to the host in its reproduction strategy. More specifically, stable coevolution becomes possible in models where only the reproduction of the host but not that of the parasite depends on the carrying capacity of the environment. From a biological perspective, a successful parasite has to rely on the host not only for replication but also for building blocks and energy. Perhaps, these results go some way to explain why, to the best of our current knowledge, no genetic parasites have ever captured neither full-fledged biosynthetic pathways, including the translation system, nor the molecular machinery for energy production.

Although the analyzed models are too simple to be of much direct relevance to extant cases of host-parasite coevolution, they are likely to be relevant for early stages of (pre) life evolution, within the RNA World, the leading current scenario for the origin of life [37–39], and at the subsequent stages, when DNA and proteins came to the scene, and different replication strategies evolved. Importantly, the replicators in this model are not abstract information carriers but self-sustaining reproducers that extract energy and building blocks from the environment, supporting both the host and the parasite [17, 35]. Conversely, the parasites neither encode their own replication machinery nor actively utilize resources, that is, their dependence on the host is complete.

The results of this work imply that primordial replicators have made innumerable “false starts” whereby host-parasite systems collapsed under the unchecked parasite pressure. Only in more evolved systems, where the capacity of the parasite to outcompete the host is balanced by defense mechanisms and, conversely, the ability of the host to eliminate the parasites is undermined by the cost of defense, stable coevolution became possible. Such coevolution between hosts and parasites is likely to be an essential driver of the evolution of biological complexity, and more specifically, of major transitions in evolution [15]. Hence any biological systems, in which a stable host-parasite coevolution regime failed to evolve, would be evolutionary dead ends.

We considered here only homogenous, well-mixed host-parasite systems. In computer simulations, compartmentalization has been shown to lead to stable co-evolutionary regimes. Most likely, compartmentalization of replicator ensembles had been part and parcel of the evolution of life from its earliest stages on, and could be considered a form of host defense, perhaps, the simplest one [18, 19]. Clearly, compartmentalization is a major path to the emergence of diversity and complexity. Further development of the models described here, in particular, by explicitly allowing evolution of the parameters defining the behavior of the parasites and replicators, will be of interest.

## Reviewers’ reports

### Reviewer 1: Olivier Tenaillon

In their manuscript “the Stability of host parasite systems: you must differ to coevolve” Faina Berezovskaya and coauthors study the existence of non trivial equilibrium in a host parasite system. The model they are interested in is the emergence of virus, transposons or mobile genetic elements. Using the existing sets of equation, the model does not allow for coexistence but rather suggests only non-invasion or the systems collapse due to parasite. TO find a range of conditions in which host and parasite can coexist they show that allowing the parasite to fully capture the energy from its host is important. This suggest that parasite must from the start be very different from their host. The problem is nicely stated and important for the current understanding of genomes that are full of mobile genetic elements. I have just some minor comments concerning how the equations could be reduced at least in the end to clearly identify the relevant biological parameters.

First, in the set of equation used K is not the capacity at equilibrium, would it be possible to have a true carrying capacity term in the equation for R and add modification in the P equation.

Response: *The terms for the environment interactions for the host and the parasite were deliberately entangled in the original formulation of the model (eq. (**1**), ref* [26]*). In this model, the host and the parasite compete for the same resources (albeit consume different amounts per individual, see more on this below). That is where the* $\frac{R+P/q}{K}$ *term comes from. The carrying capacity K, indeed, is not equal to the total population size at equilibrium. It defines the neutral point for the resource-limited replication rate rather than for the overall population growth rate. We find this usage to be quite in the spirit of the original logistic model; the alternative (defining K through the total population size at equilibrium) would entangle it with the decay term and make the equations more cumbersome. We have added the explanation to this effect to the main text.*

The impact of the q term in the logistic is not clear to me. It should be explained why q increases the speed of the parasite and decreases its contribution in the logistic. Though is makes sense qualitatively, could decoupling q in two terms solve some of the problem? Here one of the issue is that a small impact on the host resources, results in an infinite growth rate. There could be a decoupling of the two with a replication rate limit for instance, a simple saturating function could work.

Response: *The simplest way to think of the term q is to envisage a primitive system where both the replicator and the parasite are, essentially, single molecules as in the RNA world scenario. The replicator molecule encodes the complete, active replicase, whereas the parasite molecule only carries the replication recognition signals and is shorter by a factor of q. Replication of such a parasite molecule requires q times less resources and would occur q times faster, compared to the replicator molecule. We have added the explanation to this effect to the main text.*

In the final set of equation analysed (eqs. 6), here again it would be interesting to narrow down the system to its simplest form, to be able to see how general these equations are and how important are the terms linked to resistance and cost. For instance, the Parasite equation results in a pure predator equation. But the impact on the host/prey is different, mostly due to the R^2 in the replicator eq. I think discussing the similarity and reducing the final numbers of parameters could be useful for readers and for the discussion.

Response: *The R*^*2*^*term in the replicator equation and the RP term in the parasite equation both come from a simple mechanistic consideration: the entity (either the host replicator or a parasite) must encounter a replicase in order to be replicated. This is what makes this system distinct from the classic predator-prey model where the prey population growth follows the first-order kinetics (*i.e. *is not limited by the rate of intra-species encounters). We consider this feature to be an important distinction. We have added the explanation to this effect to the main text.*

A lot is spent about discussing the resistance to the parasite, but the final equation could just assume different growth rates. For the discussion, in the debate on parasite emergence, here a choice is clearly made in the equations: the parasite has to be fully dependent on its hots from the start. What if as observed in some viral system [40] there is only an initial partial dependency. This could lead to an unstable system with a prisoner’s dilemma outcome. But with a group selection pattern, the gradual emergence of parasites that can coexist with their host could emerge. For the present manuscript I just think that it could be mentioned that the present systems envision the immediate appearance of a fully dependent parasite, but that more gradual outcomes could be possible. Overall it is an interesting story.

Response: *We added the clarification that the model presumes a complete dependence of the parasite on the replicator in the model description and also in the Concluding Remarks.*

### Reviewer 2: Sandor Pongor

This is an original and significant contribution. As pointed out to the authors, I find the language difficult at times, though the writing of the paper is in general excellent and easy to follow.

Genetic parasites including mobile genetic elements, viruses and plasmids are ubiquitous among cellular organisms, and are by far the most abundant form of life. The emergence of genetic parasites is intrinsic to evolving replicator systems, but the stability of host-genetic parasite coevolution are not well understood. Berezovskaya and associates tackle this very complex problem using the method of bifurcation analysis. They convincingly show that the logically simplest model is unstable however stability can be achieved via slight modification of this model. The logics of the presentation is straightforward and easy to follow.

This reviewer finds the language too complicated at times, for instance the key sentence of the abstract “It is shown that the simplest imaginable system of this type …” appears far too complex for and abstract, and the subtitle “A highly derived genetic parasite” is hard to follow.

Response: *We appreciate the review and regret that some of the original language appeared difficult in places. We streamlined the complicated sentence in the Abstract and went through the entire manuscript to split some long sentences which, hopefully, makes the text more digestible. We presume that “subtitle” here implies the text of the respective section (the subtitle itself is very brief and straightforward). We made a few edits in this section but, overall, fail to see any source of potential confusion.*

### Reviewer 3: Alex Best

The authors present a series of models to represent dynamics between a host replicator and a genetic parasite. They show that their initial models yield no stable coexistence equilibria, but that if the parasite derives its resources purely from the host, and not the environment, then stable or cycling coexistence can occur. My background is in host-parasite coevolution in the more ‘classic’ setting of microparasites, using S-I type epidemiological models (after Kermack & McKendrick and Anderson & May). I was therefore very interested by the idea of looking at the case of genetic parasites and MGEs. The methods applied look sound and the manuscript was fairly easy to read. Overall, however, I find myself a little confused. The set-up of the ‘simplest imaginable’ model seems to me rather less an obvious starting point than their final model. This perhaps represents my background in the microparasite literature, but even so I think more could be done to explain *why* this would be an obvious starting point for a model.

The assumption of equal carrying capacities and why replicator growth is proportional to R^2 is particularly puzzling to me. I also found the figures to be of quite low print-quality, and therefore not always very helpful.

Response: *The carrying capacity in the original model is determined by the joint resource consumption whereby the replicator consumes 1 unit per individual and the parasite 1/*q *units per individual. In our model, the host replicator and the host consume the same resource and hence share a common carrying capacity although the resource consumption rates differ by a factor of q. Such a model would apply, for example, to a case when the replicator and the parasite are both molecules of the same type, such as RNA, The R*^*2*^*dynamics for the replicator and the RP dynamics for the parasite reflects the second order kinetics where the act of replication requires an encounter between a template and the replicase. We added more detailed explanations to the model description.*

Title - Could you perhaps use ‘genetic parasites’ in the title? This is a fairly specific example of a ‘host-parasite system’. Figures - The figure legends are far too brief, and the plots themselves seem to be of low quality.

Response: *Yes, fair point, we have modified the title accordingly and also emphasized genetic parasites in the revised Conclusions section of the Abstract. Several figure legends have been expanded, too.*

P5 L33 - These equations do not seem to be the obvious first step, and I’m not sure how they were reached. For example: * While it makes some sense for the parasite growth rate to be proportional to the replicator density, why is the replicator growth rate proportional to R^2? This seems particularly important as it will be this assumption that ensures the (0,0) equilibrium is always stable and this complete extinction remains possible.

* Why would we expect the parasite to be subject to the same carrying capacity, K, as the replicator?

Response: *See the explanation above.*

P5 L41 - Table 1 doesn’t seem to be here anywhere.

Response: *we regret the omission, table added.*

P5 L43 - What is ‘the template’? You haven’t defined this previously.

Response: *We use the word “template” to mean “anything that could be replicated by the replicator”. In the context of the model, it is either the replicator itself that is replicated by a replicase (which, in a simplest model of the RNA world, could be an identical molecule), or a parasite (which is incapable of producing the replicase but can serve as a template). In molecular biology, “template” is a common term when replication (or transcription) is described, so we do not expect any confusion coming out of it.*

P6 L10 - R = K is not an equilibrium of (1), as this would give dR/dt = −e_rK. I assume that is why you use the \approx symbol, but why not state the actual value.

Response: *The statement is corrected to refer to the equilibrium of a parasite-free system.*

P6 L53 - Why do you call these ‘semi-trivial’?

Response: *Because the value of one of the variables (but not both) is 0. When introducing the semi-trivial equilibrium, we indicate that it is parasite-free, so we do not believe any confusion is likely.*

P7 L31 - You have not defined these domains.

Response: *they are defined in the revised text, with reference to* Additional file 1: Mathematical Appendix 1 and Fig. 2a*.*

P7 L58 - In what sense is the parasite a ‘slightly degraded copy’ of the replicator?

Response: *we specify in the revision:* “e.g. a variant of an RNA genome that contains a deletion and thus cannot produce an active replicase”.

P9 L18 - In Fig. 4, these new coexistence equilibria seem to introduce an interesting bistability between replicator-ply and complete extinction. Could you comment on this?

Response: *Positive non-trivial equilibria A1(left) and A2 (right) (see* Fig. 4*) that appear when* $q<\frac{\left(1+e\right){e}_P}{\left(1+\alpha e\right){e}_R}$ *are both unstable: A1 is an unstable node and A2 is a saddle. Stable equilibria are only trivial (O) and semi-trivial (O2) - see* Fig. 2*, Domain 2; new stable equilibria do not appear. Unstable equilibria are not “observable” but can change the areas of attraction of stable equilibria (in particular, the area of attraction of the semi-trivial equilibrium O2 increases) and make the system orbits more complex.*

P10 L17 - As I implied above, it is a little puzzling to me why this wouldn’t be the first model you choose anyhow.

Response: *We started from the model (1) in order to show that, if the dynamics of genetic parasites is close to that of the host, then, either the parasite or the entire system goes extinct.*

Why would you assume the replicator and parasite have the same carrying capacity? This model is much closer to ‘classic’ host-parasite models.

Response: *it is not exactly the case that the carrying capacities for the replicator and the parasite are the same. Rather, K is the carrying capacity for the complete host-parasite system from which they consume resources at different rates (for more detail, see the response to Olivier Tenaillon above).*

P12 L10 - It is very interesting that you have found a limit cycle here, which does not occur in classic models. My guess is this is again to do with the R^2 in the growth term, but it would be interesting if you could explain this.

Response: *A limit cycle arises due to the term R^2 in the equation for the replicator growth that differentiates the proposed model from standard host – parasite models. As a result, the relative growth rate (dR/dt)/R is non-monotonic and has a maximum, so that the cycle arises when the null-cline intersects this maximum. Similar models describe so-called Allee-effect* [41]*. In this type of models, oscillations always arise for such parameter values where a stable equilibrium loses stability and a stable limit cycle appears around the equilibrium due to the Hopf bifurcation. This limit cycle can disappear in a heteroclinics composed by separatrixes of neighboring saddles under variation of the system parameters, and this is exactly what happened in our model (6). As a result, only unstable non-trivial equilibrium remains in the phase plane. Then, with further changing of the parameters, this equilibrium merges with the trivial equilibrium and disappears.*

## Additional files

Additional file 1: Mathematical Appendix 1. (DOCX 25 kb)

Additional file 2: Mathematical Appendix 2. (DOCX 16 kb)

Additional file 3: Mathematical Appendix 3. (DOCX 28 kb)

Additional file 4: Mathematical Appendix 4. (DOCX 19 kb)

## Abbreviation

MGE: Mobile genetic elements
