# Supplementary material for: Stable coevolutionary regimes for genetic parasites and their hosts: you must differ to coevolve
Source: Biol Direct. 2018 Dec 14;13:27. doi: 10.1186/s13062-018-0230-9 (PMC6822691; doi:10.1186/s13062-018-0230-9)
Supplement: Supplementary file 1 — Mathematical Appendix 1. (DOCX 25 kb) [file 13062_2018_230_MOESM1_ESM.docx]

**Mathematical Appendix 1. Equilibria and bifurcation diagram of model (1) :**

$\frac{dR}{dt}=\frac{1}{1+\alpha e}R^{2}\left( 1-\frac{R+\frac{P}{q}}{K} \right){-e}_{R}R\equiv F_{R}\left( R,P \right),$ (1)

$$\frac{dP}{dt}=\frac{q}{1+e}RP\left( 1-\frac{R+\frac{P}{q}}{K} \right)-e_{P}P\equiv F_{P}(R,P)$$

**1. Null-isoclines**

Consider null-isoclines of the model (1), which, by definition, are defined by equations ${dR/dt\equiv F}_{R}\left( R,P \right)=0$and ${dP/dt\equiv F}_{P}\left( R,P \right)=0.$ The first equation defines trivial null-isocline $R=0$ and non-trivial null-isocline given by the equation

$\frac{1}{1+\alpha e}R\left( 1-\frac{R+\frac{P}{q}}{K} \right){-e}_{R}=0$ . (A1.1)

The second equation $F_{P}\left( R,P \right)=0$ defines trivial null-isocline $P=0$ and non-trivial null-isocline given by the equation

$\frac{q}{1+e}R\left( 1-\frac{R+\frac{P}{q}}{K} \right)-e_{P}=0.$ (A1.2)

Solving (A1.1) with respect to$P$ we get expression for null-isocline $P=P_{1}\left( R \right)$:

$P_{1}\left( R \right)=\frac{q(R\left( K-R \right)-e_{R}K\left( 1+\alpha e \right))}{R}$ (A1.3)

Solving (A1.2) with respect to$P$ we get expression for null-isocline $P=P_{2}\left( R \right)$:

$P_{2}\left( R \right)=\frac{q R\left( K-R \right)-e_{P}K\left( 1+e \right)}{R}$ (A1.4)

Non-trivial equilibria of system (1), i.e. those that have both non-zero coordinates, are the points of intersection of curves (A1.3) and (A1.4). We see however that expressions for the curves $P_{1}\left( R \right)$ and $P_{2}\left( R \right)$ have similar form and differ only by the values of coefficients $q\left( 1+\alpha e \right)e_{R}$, $\left( 1+e \right)e_{P}$. Typical shapes of these isoclines in the case $q\left( 1+\alpha e \right)e_{R} \neq\left( 1+e \right)e_{P}$ , are shown in Fig.1.

Notice, that for $\left( 1+\alpha e \right)e_{R}q=\left( 1+e \right)e_{P},$ i.e. for

$q=\frac{\left( 1+e \right)e_{P}}{\left( 1+\alpha e \right)e_{R}}$ (A1.5)

the lines $P_{1}$ and $P_{2}$coincide. Hence, in this case system (1) has a line $P=P(R)$ *of non-isolated equilibria.* Excluding parameter *q* we can write the expression for this line as

$P=\frac{(R\left( K-R \right)-e_{R}K\left( 1+\alpha e \right))(1+e)e_{P}}{\left( 1+\alpha e \right)e_{R}R}$ (A1.6)

Plots of isoclines (A1.3), (A1.4) and (A1.6) are shown in Fig.1.

**2. Non-trivial Equilibria**

Coordinates of equilibrium points satisfy to the system

$F_{R}\left( R,P \right)=0, F_{P}\left( R,P \right)=0$ . (A1.7)

It is easily to see that the system always has the trivial equilibrium $O(R=0,P=0)$; a simple algebra shows that the system also has equilibria

$O_{1}(R_{1}=\frac{K-\sqrt{K(K-4(1+\left( 1+\alpha e \right)e_{R})}}{2}$, $P=0),$ $O_{2}\left( R_{2}=\frac{K+\sqrt{K(K-4(1+\left( 1+\alpha e \right)e_{R})}}{2},P=0 \right)$ (A1.8)

if

$4(1+\left( 1+\alpha e \right)e_{R})<K$ . (A1.9)

The equilibria $O_{1}, O_{2}$ appear/disappear in the phase plane $\left( R,P \right)$ if

$e=\frac{K-4e_{R}}{4\alpha e_{R}}$ . (A1.10)

In this case $O_{1}= O_{2}=\left( \frac{K}{2},0 \right).$If$4(1+\left( 1+\alpha e \right)e_{R})>K$ then the model has only trivial equilibrium *O*.

*Stability* of equilibria can be investigated by standard methods of linearization (see, e.g., Andronov et. al.,1973; Blanchard et.al., 2011). Let $J\left( R,P \right)$ be Jacobian of system (1):

$J\left( R,P \right)=\left( \begin{matrix} \frac{\partial\left( F_{R} \right)}{\partial R} & \frac{\partial\left( F_{R} \right)}{\partial P} \\ \frac{\partial\left( F_{P} \right)}{\partial R} & \frac{\partial\left( F_{P} \right)}{\partial P} \end{matrix} \right)\equiv\left( \begin{matrix} a_{11} & a_{12} \\ a_{21} & a_{22} \end{matrix} \right)$ (A1.11)

where

$a_{11}=\frac{1}{1+\alpha e}(2R\left( 1-\frac{R+\frac{P}{q}}{K} \right)-\frac{R^{2}}{K}){-e}_{R},$ $a_{12}=-\frac{R^{2}}{\left( 1+\alpha e \right)Kq},$

$a_{21}=(\frac{Pq}{1+e}\left( 1-\frac{R+\frac{P}{q}}{K} \right)-\frac{R}{K}),$ $a_{22}=(\frac{1}{1+e}(qR\left( 1-\frac{R+\frac{P}{q}}{K} \right)-\frac{PR}{K}){-e}_{P},$

For $P=0$ the entries of the Jacobian are:

$a_{11}=\frac{1}{1+\alpha e}(2R\left( 1-\frac{R}{K} \right)-\frac{R^{2}}{K}){-e}_{R},$ $a_{12}=-\frac{R^{2}}{\left( 1+\alpha e \right)Kq},$ (A1.12)

$a_{21}=0,$ $a_{22}=\frac{1}{1+e}(qR\left( 1-\frac{R}{K} \right)){-e}_{P}$.

Substituting $R=0, R=R_{1}, R=R_{2}$ to (A1.12) we get expressions for eigenvalues $\mu$ of equilibrium points $O, O_{1}, O_{2}$.

***Proposition* 1.**

1. *The eigenvaluesof equilibrium point* $O$ *are*

$\mu_{1}\left( O \right)={-e}_{R}, \mu_{2}\left( O \right)={-e}_{P}$*.* (A1.13)

1. *If condition (A1.9) holds then*
2. *the eigenvaluesof equilibrium point*$O_{1}$*are*

$\mu_{1}\left( O_{1} \right)=\frac{\sqrt{(K-4\left( 1+\left( 1+\alpha e \right)e_{R} \right)}(\sqrt{K}-\sqrt{(K-4\left( 1+\left( 1+\alpha e \right)e_{R} \right)}}{2\left( 1+\alpha e \right)}>0 ,$(A1.14)

$\mu_{2}\left( O_{1} \right)=\frac{q\left( 1+\alpha e \right)e_{R}-\left( 1+e \right)e_{P}}{\left( 1+e \right)e_{R}}$*;* (A1.15)

1. *the eigenvaluesof equilibrium point*$O_{2}$ *are*

$\mu_{1}\left( O_{2} \right)=-\frac{\sqrt{(K-4\left( 1+\left( 1+\alpha e \right)e_{R} \right)}(\sqrt{K}+\sqrt{(K-4\left( 1+\left( 1+\alpha e \right)e_{R} \right)}}{2\left( 1+\alpha e \right)}<0 ,$(A1.16)

$\mu_{2}\left( O_{2} \right)=\frac{q\left( 1+\alpha e \right)e_{R}-\left( 1+e \right)e_{P}}{\left( 1+e \right)e_{R}}$*.* (A1.17)

***Corollary 1.*** *The trivial equilibrium* $O$ *is a stable node for all parameter/coefficient values of system* (1).

Notice that if condition (A1.10) holds then the equilibrium $O_{12}\left( R_{12}=\frac{K}{2},P=0 \right)$ has the eigenvalue $\mu_{1}\left( O_{12} \right)=$0. Hence, equality (A1.10) defines the condition of degeneracy for model (1) (fold bifurcation, see Kuznetsov, 1995). If $e<\frac{K-4e_{R}}{4\alpha e_{R}}$ then the model has three equilibria $O,O_{1}, O_{2}$ and has only trivial equilibrium *O* if $e>\frac{K-4e_{R}}{4\alpha e_{R}}$ .

Let now $e<\frac{K-4e_{R}}{4\alpha e_{R}}.$Then the second eigenvalues of equilibria $O_{1}, O_{2}$ are equal:

$\mu_{2}\left( O_{1} \right)=\mu_{2}\left( O_{2} \right)\equiv\mu_{2}=\frac{q\left( 1+\alpha e \right)e_{R}-\left( 1+e \right)e_{P}}{\left( 1+e \right)e_{R}}.$

It is easily to verify that $\mu_{2}>0$ if $q>\frac{\left( 1+e \right)e_{P}}{\left( 1+\alpha e \right)e_{R}}$ and $\mu_{2}<0$ if $q<\frac{\left( 1+e \right)e_{P}}{\left( 1+\alpha e \right)e_{R}}$; $\mu_{2}=0$ if equality (A1.5) holds: $q=\frac{\left( 1+e \right)e_{P}}{\left( 1+\alpha e \right)e_{R}}$.

Recall that in the last case model (1) has the line of non- isolated equilibrium points (A1.6).

Now we can formulate the properties of equilibria $O_{1}, O_{2}$ in general cases.

***Proposition 2.*** *If condition (A1.9) holds then*

1. *equilibrium* $O_{1}$*is a saddle and* $O_{2}$ *is a stable node* if $q<\frac{\left( 1+e \right)e_{P}}{\left( 1+\alpha e \right)e_{R}}$;
2. *equilibrium* $O_{1}$*is an unstable node and* $O_{2}$ *is a saddle* if $q>\frac{\left( 1+e \right)e_{P}}{\left( 1+\alpha e \right)e_{R}}$ (see Fig.2).

1. **Bifurcation Diagram and Phase Portraits**

Now we can describe the bifurcation diagram (phase-parameter portrait) of model (1) taking as parameters $q$ and *e* supposing that the values of all other coefficients of the model are fixed, see Figure 2.

**Theorem 1.**

*Let* $e$ *and* $q$ *be the parameters of model (1) and* $\alpha,K,e_{R}, e_{P}$ *be arbitrary values of fixed coefficients. Positive parameter space {e, q} is dividing into 3 domains* ***D.1, D.2, D.3*** *of qualitatively (topologically) different phase portraits of the model in the first quadrant of the plane* $\left( R,P \right)$ *(see Fig.2a, b).*

*1) With parameter values belonging to Domain 1* $\left\{ e>\frac{K-4e_{R}}{4\alpha e_{R}} \right\}$*the model has a single stable equilibrium O and has no other attractors;*

*2) With parameter values belonging to Domain 2* $\{ e<\frac{K-4e_{R}}{4\alpha e_{R}}, q<\frac{\left( 1+e \right)e_{P}}{\left( 1+\alpha e \right)e_{R}}\}$ *the model has three equilibria,* ${O,O}_{1}{,O}_{2}$*. Here* $O, O_{2}$ *are stable nodes and* $O_{1}$ *is a saddle which divides areas of attraction of* $O, O_{2}$ *for small values of* $P$*;*

*3) with parameter values belonging to Domain 3* $\{ e<\frac{K-4e_{R}}{4\alpha e_{R}}, q>\frac{\left( 1+e \right)e_{P}}{\left( 1+\alpha e \right)e_{R}}\}$ *the model also has three equilibria,* ${O,O}_{1}{,O}_{2}$*. Here* $O$*is a stable node,* $O_{1}$*is an unstable node and* $O_{2}$ *is a saddle;* $O$ *is unique attractor in the first quadrant of plane* $\left( R,P \right).$

*4) The line* $\boldsymbol{B:}e=\frac{K-4e_{R}}{4\alpha e_{R}}$ *serves as the boundary between Domain 1 and Domains 2,3. In this boundary the phase portrait of the system contains the equilibrium O, which is a stable node, and saddle-node equilibrium* $O_{12}\left( R_{12}=\frac{K}{2},P=0 \right)$*if* $q\neq\frac{\left( 1+e \right)e_{P}}{\left( 1+\alpha e \right)e_{R}}$ *(see Fig.3, left panel);*

*The line* $\boldsymbol{Q:}q=\frac{\left( 1+e \right)e_{P}}{\left( 1+\alpha e \right)e_{R}}$*serves as the boundary between Domains 2 and 3. At this boundary the phase portrait of the system contains a stable equilibrium* $O$ *and the curve of non-isolated equilibria* $P=\frac{(R\left( K-R \right)-e_{R}K\left( 1+\alpha e \right)(1+e)e_{P}}{\left( 1+\alpha e \right)e_{R}R}$ *(see Fig.3, right panel). The points* $\left( R,P \right)$ *of this curve are repelling from the left side of the curve and attractive from the right side.*

.

***Corollary 2.*** Model (1) demonstrates only two asymptotically different phase behaviors with parameter values that does not belong to the boundaries ***B*** and ***Q***: monostability with equilibrium ***O*** in the union of Domains ***D.1*** and ***D.3*** and bistability with equilibria ***O*** and ***O_2_*** in Domain 2, where the final behavior depends on initial values of *P* and *R*.
