# Supplementary material for: Stable coevolutionary regimes for genetic parasites and their hosts: you must differ to coevolve
Source: Biol Direct. 2018 Dec 14;13:27. doi: 10.1186/s13062-018-0230-9 (PMC6822691; doi:10.1186/s13062-018-0230-9)
Supplement: Supplementary file 2 — Mathematical Appendix 2. (DOCX 16 kb) [file 13062_2018_230_MOESM2_ESM.docx]

**Mathematical Application 2. Model (1) with Additional interactions of replicators *R* and parasites *P***

If to assume that parasites decrease replicator growth rate, then the model (1) takes the form

$\frac{dR}{dt}=\frac{1}{1+\alpha e}R^{2}\left( 1-\frac{R+\frac{P}{q}}{K} \right)-bPR{-e}_{R}R\equiv F_{R}\left( R,P \right),$ (A2.1)

$$\frac{dP}{dt}=\frac{q}{1+e}RP\left( 1-\frac{R+\frac{P}{q}}{K} \right)-e_{P}P\equiv F_{P}(R,P)$$

where $b$ is a (small) positive parameter.

Our analysis shows that equilibria (A1.8) $O$, $O_{1}, O_{2}$ with $P=0$ still exist in system (A2.1) under the same conditions as for system (1). Additionally, new equilibria $A_{1}\left( P^{*},R^{+} \right), A_{2}(P^{*},R^{-})$ can appeare/disappeare in model (A2.1) (see Fig.4a), where

$P^{*}=\frac{(1+e)e_{P}-q\left( 1+\alpha e \right)e_{R}}{bq\left( 1+\alpha e \right)},$ (A2.2)

${\begin{aligned} \\ R \end{aligned}}^{+,-}=\frac{q\left( 1+\alpha e \right)e_{R}-\left( 1+e \right)e_{P}+bKq^{2}(1+\alpha e)\pm\sqrt{{((1+e)e_{P}-(1+\alpha e)q(e_{R}+bKq))}^{2}-4(1+e)b^{2}{(1+\alpha e)}^{2}e_{P}Kq^{3}}}{2a(1+\alpha e)q^{2}}$ .

According to formula (A2.2), $P^{*}>0$only if $q<\frac{\left( 1+e \right)e_{P}}{\left( 1+\alpha e \right)e_{R}}$ . For $q=\frac{\left( 1+e \right)e_{P}}{\left( 1+\alpha e \right)e_{R}}$ the points $A_{1}{,A}_{2}$ coincide with the points $O_{1}{,O}_{2}$, correspondingly.

A structure of equilibrium can be defined by the determinant and trace of their Jacobian.

**Proposition 3.** *If* $q<\frac{\left( 1+e \right)e_{P}}{\left( 1+\alpha e \right)e_{R}}$ *then equilibrium* $A_{1}$ *is an unstable node and* $A_{2}$ *is a saddle (see Fig 4a). If* $q<\frac{\left( 1+e \right)e_{P}}{\left( 1+\alpha e \right)e_{R}}$ *then the model (A2.1) has no* *positive nontrivial equilibria (see Fig 4b)*.

Proof.

Determinant and trace of the Jacobian of system (A2.1) at the points $A_{1}\left( P^{*},R^{+} \right), A_{2}(P^{*},R^{-})$ are equal to

*DJ*$(A_{1},A_{2})$=$\frac{b(-P^{*}+q(K-2R^{-,+}))}{(1+e)K}$, *TrJ*= $\frac{-P^{*}(1+e+q\left( 1+\alpha e \right))+(1+e)q(K-2R^{-,+})}{(1+e)(1+\alpha e)Kq}$.

One can write the values $R^{+,-}$in formula (A2.2) in the form

${R^{-}=\frac{1}{2q}\left( \left( Kq-P \right)+\sqrt{D} \right), R}^{+}=\frac{1}{2q}(\left( Kq-P \right)-\sqrt{D})$ (A2.3)

where $D=\left( Kq-P \right)^{2}-4e_{P}Kq\left( 1+e \right)<\left( Kq-P \right)^{2}.$ Then a simple algebra shows that

$DJ\left( A_{1} \right)=\frac{b\sqrt{D}P}{K(1+e)}>0,DJ\left( A_{2} \right)=-\frac{b\sqrt{D}P}{K\left( 1+e \right)}<0$ . So, the point $A_{2}(P^{*},R^{+})$ is a saddle and $A_{1}(P^{*},R^{-})$ is an unstable node (because *TrJ(*$A_{1})>0)$*.*

Q.i.d.

Figure 4b allows to compare the phase portraits of the model when $b>0$ and $b=0$*.*

It follows from Proposition 3 and Figures 4a, b that although model (A2.1) with additional interactions between replicators and parasites has more rich and complex phase portraits, in particular, may have non-trivial equilibria with non-zero values of both coordinates, these additional equilibria are unstable.
