# Supplementary material for: Stable coevolutionary regimes for genetic parasites and their hosts: you must differ to coevolve
Source: Biol Direct. 2018 Dec 14;13:27. doi: 10.1186/s13062-018-0230-9 (PMC6822691; doi:10.1186/s13062-018-0230-9)
Supplement: Supplementary file 3 — Mathematical Appendix 3. (DOCX 28 kb) [file 13062_2018_230_MOESM3_ESM.docx]

**Mathematical Appendix 3**

**“Volterra”- type Modification of Model (1)**

1. **Modified model; isoclines and equilibria**

We have shown that model (1) does not show stable equilibria or regimes of coexistence of replicators and genetic parasites; in contrast, only parasite-free state in general can be evolutionary stable in this model. A possible reason of this phenomenon is as follows.

Let us introduce a new variable $x(t)$ by the following equation:

$\frac{dx}{dt}= R\left( 1-\frac{R+\frac{P}{q}}{K} \right)$, $x\left( 0 \right)=0.$

Then model (1) can be written in the form

$\frac{dR}{dt}=R(\frac{1}{1+\alpha e}\frac{dx}{dt} {-e}_{R})$, (A3.1)

$\frac{dP}{dt}=P(\frac{q}{1+e}\frac{dx}{dt}-e_{P})$.

The solution to this system is given by the formulas

$R\left( t \right)=R\left( 0 \right)\exp(\frac{1}{1+\alpha e}{x(t)-e}_{R}t)$, (A3.2)

$P\left( t \right)=P\left( 0 \right)\exp(\frac{q}{1+e}x(t){-e}_{P}t)$.

We can see now that the dynamics of replicators and parasites are described by equations of ***identical form.*** The difference is only in the values of coefficients.

It is naturally to assume that the dynamics of replicators and parasites should be qualitatively different; this assumption points the way to modify the model. Let us consider the following modification of model (1) where dynamics of parasite is described by “Volterra-type” equation:

$\frac{dR}{dt}=\frac{1}{1+\alpha e}R^{2}\left( 1-\frac{R+\frac{P}{q}}{K} \right){-e}_{R}R\equiv F_{R}\left( R,P \right),$ (A3.3)

$\frac{dP}{dt}=\frac{q}{1+e}RP-e_{P}P\equiv G_{P}(R,P)$.

**Remark.**  Various modifications of Volterra model were described in Bazykin (2000), see also Bazykin, Berezovskaya (1995).

Null-isoclines of model (A3.3) are defined by the equations

$F_{R}\left( R,P \right)=0 ,G_{P}\left( R,P \right)=0$ . (A3.4)

The first equation defines trivial null-isocline $R=0$ and non-trivial null-isocline

$P\left( R \right)=\frac{q\left( R\left( K-R \right)-e_{R}K\left( 1+\alpha e \right) \right)}{R}$ . (A3.5)

The second equation $G_{P}\left( R,P \right)=0$ defines trivial null-isocline $P=0$ and non-trivial null-isocline

$R=\left( 1+e \right)e_{P}/q$ . (A3.6)

Figure 5 shows that system (A3.3) can have the same equilibria with *P*=0 as model (1) and additionally *nontrivial* *equilibrium*.

More precisely, model (A3.3) always has equilibrium $O\left( R=0,P=0 \right),$ may have equilibria

$O_{1}\left( R_{1}=\frac{K-\sqrt{K(K-4(1+\left( 1+\alpha e \right)e_{R})}}{2},P=0 \right)$, $O_{2}(R_{2}=\frac{K+\sqrt{K(K-4(1+\left( 1+\alpha e \right)e_{R})}}{2}$, $P=0)$

if $K-4(1+\left( 1+\alpha e \right)e_{R})\geq0$, that is if $e<(K-4\left( 1+e_{R} \right))/(4\alpha e_{R})$ ,

and has equilibrium $A\left( R_{A},P_{A} \right)$where

$R_{A}=\frac{(1+e)e_{p}}{q},P_{A}=\frac{Kq(1+e)e_{p}-{(1+e)}^{2}{e_{p}}^{2}-(1+\alpha e)e_{R}Kq^{2}}{(1+e)e_{p}}$ . (A3.7)

The equilibrium $A\left( R_{A},P_{A} \right)$ may be parasite-free at some parameter values; to find these parameters, let us put $P_{A}=0$in (A3.7). We get two branches $q_{1,2}=q\left( e,K, \alpha, e_{P},e_{R} \right):$

$q_{1}:q=\frac{\left( 1+e \right)e_{p}\left( K+\sqrt{K\left( K-4\left( 1+\alpha e \right)e_{R} \right)} \right)}{2\left( 1+\alpha e \right)e_{R}K}$, $q_{2}:q=\frac{\left( 1+e \right)e_{p}\left( K-\sqrt{K\left( K-4\left( 1+\alpha e \right)e_{R} \right)} \right)}{2\left( 1+\alpha e \right)e_{R}K}$ . (A3.8)

For these $q$ the coordinate $P_{A}=0$ and the coordinate $R_{A}$ becomes

${R_{A}}^{1}=\frac{2\left( 1+\alpha e \right)K}{K+\sqrt{K\left( K-4\left( 1+\alpha e \right)e_{R} \right)}}=\frac{1}{2}\left( K-\sqrt{K(K-4\left( 1+\alpha e \right)e_{R}} \right)=R_{1}$ , (A3.9)

${R_{A}}^{2}=\frac{2\left( 1+\alpha e \right)e_{R}K}{K+\sqrt{K\left( K-4\left( 1+\alpha e \right)e_{R} \right)}}=\frac{1}{2}\left( K+\sqrt{K(K-4\left( 1+\alpha e \right)e_{R}} \right)=R_{2}$ (A3.10)

So, we have proven the following

**Proposition 4.** *For parameter values belonging to the parameter branch* $q_{1}$ *the equilibrium* $A\left( R_{A},P_{A} \right)$ *coincides with equilibrium* $O_{1}(R_{1}=\frac{K-\sqrt{K(K-4\left( 1+\alpha e \right)e_{R})}}{2}$*,* $P=0);$ *for parameter values belonging to the parameter branch* $q_{2}$ *the equilibrium* $A\left( R_{A},P_{A} \right)$ *coincides with equilibrium* $O_{2}(R_{2}=\frac{K-\sqrt{K(K-4\left( 1+\alpha e \right)e_{R})}}{2}$*,* $P=0).$ *For positive* $e$ *the branches* $q_{1}$ *and* $q_{2}$*coincides under condition (A1.10), i.e. when* $K=4\left( 1+\alpha e \right)e_{R}$ *or, equivalently, when* $e=\frac{K-4e_{R}}{4\alpha le_{R}}$*.*

The branches $Q_{1}$ and $Q_{2}$ bound a domain of parameter values (see Fig.6a below) in which there exists a non-trivial equilibrium $A\left( R_{A},P_{A} \right)$ with non-zero values of both coordinates.

**2. Stability of equilibria and bifurcation diagram**

For analysis of stability of equilibria of system (A3.3) let us consider it’s Jacobian

$J_{V}\left( R,P \right)=\left( \begin{matrix} \frac{\partial\left( F_{R} \right)}{\partial R} & \frac{\partial\left( F_{R} \right)}{\partial P} \\ \frac{\partial\left( G_{P} \right)}{\partial R} & \frac{\partial\left( G_{P} \right)}{\partial P} \end{matrix} \right)\equiv\left( \begin{matrix} b_{11} & b_{12} \\ b_{21} & b_{22} \end{matrix} \right)$ (A3.11)

where

$b_{11}=\frac{R(2Kq-2P-3qR)}{\left( 1+\alpha e \right)Kq}-e_{R},$ $b_{12}=-\frac{R^{2}}{\left( 1+\alpha e \right)Kq},$ $b_{21}=\frac{Pq}{1+e},$ $b_{22}=-e_{P}+\frac{qR}{1+e}$.

If $P=0$ than the entries of the Jacobian are:

$b_{11}=\frac{1}{1+\alpha e}\frac{R(2K-3R)}{K}{-e}_{R},$ $b_{12}=-\frac{R^{2}}{\left( 1+\alpha e \right)Kq},$ $b_{21}=0,$ $b_{22}=\frac{qR}{1+e}{-e}_{P}.$

Substituting $R=0, R=R_{1}, R=R_{2}$ to (A3.12) we can compute the eigenvalues $\mu$ of equilibrium points $O, O_{1}, O_{2}$.

Let us reformulate the condition (A1.9), $4(1+\left( 1+\alpha e \right)e_{R})<K$ , as

$e<\frac{K-4\left( 1+e_{R} \right)}{4\alpha e_{R}}$. (A3.12)

***Proposition* 5*.***

*a) The eigenvaluesof equilibrium point* $O$ *are*

$\mu_{1}\left( O \right)={-e}_{R}, \mu_{2}\left( O \right)={-e}_{P} .$ (A3.13)

*b) If condition (A3.12) holds then eigenvaluesof equilibrium* $O_{1}$ *are*

$\mu_{1}\left( O_{1} \right)=\frac{\sqrt{(K-4\left( 1+\left( 1+\alpha e \right)e_{R} \right)}(\sqrt{K}-\sqrt{(K-4\left( 1+\left( 1+\alpha e \right)e_{R} \right)}}{2\left( 1+\alpha e \right)}>0$(A3.14)

$\mu_{2}\left( O_{1} \right)={-e}_{P}+\frac{(K-\sqrt{K(K-4(1+\alpha e)e_{R})})q}{2(1+e)}$, (A3.15)

*c) If condition (A3.12) holds then the eigenvaluesof equilibrium* $O_{2}$*are*

$\mu_{1}\left( O_{2} \right)=-\frac{\sqrt{(K-4\left( 1+\left( 1+\alpha e \right)e_{R} \right)}(\sqrt{K}+\sqrt{(K-4\left( 1+\left( 1+\alpha e \right)e_{R} \right)}}{2\left( 1+\alpha e \right)}<0$(A3.16)

$\mu_{2}\left( O_{2} \right)={-e}_{P}+\frac{(K+\sqrt{K(K-4(1+\alpha e)e_{R})})q}{2(1+e)}$*.* (A3.17)

***Corollary 1.*** *Equilibrium point* $O$ *is a stable node for all parameter/coefficient values of system (A3.3).*

Substituting coordinates of the point *A* to (A3.11) we obtain the following entries of Jacobian $J_{V}\left( R,P \right)$:

$b_{11}=e_{R}-\frac{\left( 1+e \right)^{2}e_{p}^{2}}{K(1+\alpha e)q^{2}}, b_{12}=-\frac{\left( 1+e \right)^{2}e_{p}^{2}}{K(1+\alpha e)q^{3}},$ (A3.18)

$b_{21}=q\left( -e_{P}+\frac{Kq}{1+e}-\frac{\left( 1+\alpha e \right)e_{R}Kq^{2}}{\left( 1+e \right)^{2}e_{p}} \right),b_{22}=0$.

Determinant and trace of the Jacobian in the point $A$ are

$Det(J_{V}\left( R_{A},P_{A} \right)) =\frac{e_{p}((1+e)e_{p}Kq-(1+\alpha e)e_{R}Kq^{2}-{(1+e)}^{2}e_{p}^{2})}{(1+\alpha e){Kq}^{2}}$, (A3.19)

$Tr(J_{V}\left( R_{A},P_{A} \right))=e_{R}-\frac{{(1+e)}^{2}e_{p}^{2}}{(1+\alpha e)Kq^{2}}$ (A3.20)

$Det\left( J_{V}\left( R_{A},P_{A} \right) \right)=0$when

$q=\frac{\left( 1+e \right)e_{p}\left( K\pm\sqrt{K\left( K-4\left( 1+\alpha e \right)e_{R} \right)} \right)}{2\left( 1+\alpha e \right)e_{R}K}$ (A3.21)

and $Det\left( J_{V}\left( R_{A},P_{A} \right) \right)>0$ when

$q_{1}<q<q_{2}$ (A3.22)

where $q_{1}= \frac{\left( 1+e \right)e_{p}\left( K-\sqrt{K\left( K-4\left( 1+\alpha e \right)e_{R} \right)} \right)}{2\left( 1+\alpha e \right)e_{R}K}$, $q_{2}=\frac{\left( 1+e \right)e_{p}\left( K+\sqrt{K\left( K-4\left( 1+\alpha e \right)e_{R} \right)} \right)}{2\left( 1+\alpha e \right)e_{R}K}$.

Thus, equilibrium $A$ is non-saddle (node or spiral) if $A$ is positive and $q$ satisfies (A3.22). Notice that for these $q$ the points $O_{1}, O_{2}$ are saddles.

Trace $Tr\left( J_{V}\left( R_{A},P_{A} \right) \right)=0$ on the curve $Tr: q=\frac{\left( 1+e \right)e_{p}}{\sqrt{\left( 1+\alpha e \right)e_{R}K}}$. Equilibrium$A$ changes stability when parameters cross this curve. Changing stability of $A$ corresponds, generally, to the Andronov-Hopf bifurcation (see, e.g., Kuznetzov 1998) that happens when $Tr\left( J_{V}\left( R_{A},P_{A} \right) \right)=0$. This bifurcation is supercritical (a stable limit cycle appears) for a wide range of the model parameters.

To prove the last statement, we use the following statement that follows from the formulas given in (Bautin, Leontovich, Ch.11, s.5, 1975): the sign of the first Lyapunov value $l_{1}$ at the curve *Tr* coinsides with the sign of expression

$l=\left( \frac{2\left( 1+e \right)^{4}e_{p}^{2}}{q}-\left( K\left( 1+\alpha e \right) \right)^{2}q^{3}-\left( 1+e \right)^{2}e_{P}K\left( \left( 1+e \right)e_{P}-\left( 1+\alpha e \right)q \right) \right).$

Our computations of $l$ for a wide range of parameters showed that$l$ < 0, so $l_{1}$ <0 . Thus, we have the following statement.

**Proposition 6.** *Non-trivial*  *equilibrium* $A\left( R_{A},P_{A} \right)$ *of system (A3.3) where* $R_{A},P_{A}$ *are given by formulas (A3.7) is non-saddle and may be a stable/unstable node, spiral or center (see Fig.5) in the area of its positivity bounded by closed curves (A3.8).* *Equilibrium* $A$ *is stable in Domain 4 and unstable in Domains 5 and 6; in Domain 5 the point A is placed inside the stable limit cycle.*

Now we can describe the phase-parameter portrait of model (A3.3).

**Theorem 2.** *Positive parameter space* $\{q,e\}$ *at fixed positive coefficients*$\alpha,K,e_{R}, e_{P}$*is dividing into 6 Domains of qualitatively (topologically) different phase portraits of the model (A33) for positive* $\left( R,P \right)$*.*

*Domain 1 contains only one equilibrium O.*

*Domains 2, 3 contain three equilibria* $O,O_{1}, O_{2}.$

*Domains 4,5,6 contain four equilibria* $O,O_{1}, O_{2}$*, and* $A.$

*Equilibrium point O exists and is stable in all Domains; it is the only attractor in Domains 1,3,6.*

*There exist two attractors in Domains 2, 4, 5, namely, the points O,* $O_{2}$ *in Domain 2, the points O,* $A$ *in Domain 4, the point O and the stable limit cycle in Domain 5; the separatrix of saddle* $O_{1}$*divides areas of attractions of the point O and the stable limit cycle for small enough P.*

*The following curves serve as the boundaries of* $\{q,e\}$*-parameter portrait of system (A3.3):*

$q_{1}:q(e)=\frac{\left( 1+e \right)e_{p}\left( K+\sqrt{K\left( K-4\left( 1+\alpha e \right)e_{R} \right)} \right)}{2\left( 1+\alpha e \right)e_{R}K}{, q}_{2}:q(e)=\frac{\left( 1+e \right)e_{p}\left( K-\sqrt{K\left( K-4\left( 1+\alpha e \right)e_{R} \right)} \right)}{2\left( 1+\alpha e \right)e_{R}K}$*,*

$Tr: q(e)=\frac{(1+e)e_{p}}{\sqrt{\left( 1+\alpha e \right)e_{R}K}}$ *,* $B:e= \frac{K-4e_{R}}{4\alpha e_{R}}$ *, L* (A3.21)

*where* $q_{1}\cup q_{2}$ *is the boundary of the union of domains where the equilibrium* $A$ *is positive, the boundary* $Tr$ *:* $Tr\left( J_{V}\left( R_{A},P_{A} \right) \right)=0$*corresponds to changing of stability of* $A$*; the boundary B corresponds to appearance/disappearance of* $O_{1}, O_{2}$*; the curve L corresponds to appearance/disappearance of separatrix cycle composed by separatrices of saddles* $O_{1}, O_{2};$ *a stable limit cycle, which exists in Domain 5 disappears on the boundary L.*

The bifurcation diagram and phase portraits of the model are presented in Fig-s.6a, b.

In Fig.6a the boundaries between domains of qualitatively different behaviors of the system are the curves given by formulas (A3.21):

the curve ${q_{1,2}:q}_{1}\cup q_{2}$ bound the close area (containing domains D4, D5, D6 inside) in which the system has non-trivial equilibrium $A$ as well as semi-trivial parasite-free equilibria $O_{1,2};$

the curve $Tr$is the boundary between domains D4, D5; it corresponds to changing stability of equilibrium $A$ via Andronov-Hopf bifurcation accompanied by appearance of a limit cycle;

the curve *L* is the boundary between domains D5, D6; it corresponds to disappearance of the limit cycle in the heteroclinics of the saddle equilibria $O_{1}, O_{2}$ (notice, that the curve *L* corresponds to non-local bifurcation and has no analytic description);

the curve $B$ is the boundary between domains D1, D2 and D3; it corresponds to appearing/disappearing of the equilibrium $O_{1,2}$ in the phase plane $(R,P)$.

Qualitatively portraits of system (A3.3) in domains D1, D2, D3 are the same as the portraits given in Fig.2 for system (1).
