# Supplementary material for: Stable coevolutionary regimes for genetic parasites and their hosts: you must differ to coevolve
Source: Biol Direct. 2018 Dec 14;13:27. doi: 10.1186/s13062-018-0230-9 (PMC6822691; doi:10.1186/s13062-018-0230-9)
Supplement: Supplementary file 4 — Mathematical Appendix 4. (DOCX 19 kb) [file 13062_2018_230_MOESM4_ESM.docx]

**Mathematical Appendix 4.**

**Volterra-like model in case when *parasites decrease the growth rate of replicators***

In this case the model (A3.3) takes the form

$\frac{dR}{dt}=\frac{1}{\left( 1+\alpha e \right)}R^{2}\left( 1-\frac{R+\frac{P}{q}}{K} \right)-bPR{-e}_{R}R\equiv F_{R}\left( R,P,a \right),$ (A4.1)

$$\frac{dP}{dt}=\frac{q}{\left( 1+e \right)}RP-e_{P}P\equiv G_{P}(R,P)$$

where $b$ is (small) positive parameter; the term -$bPR$ in the first equation reflects decreasing of a replicator growth rate due to parasites.

Similar to system (A3.3) the system (A4.1) always has trivial equilibrium $O\left( R=0,P=0 \right)$ and can have semi-trivial equilibria $O_{1}(R_{1}=\frac{K-\sqrt{K(K-4(1+\left( 1+\alpha e \right)e_{R})}}{2}$, $P=0),$

$O_{2}\left( R_{2}=\frac{K+\sqrt{K(K-4(1+\left( 1+\alpha e \right)e_{R})}}{2},P=0 \right)$ if $K-4(1+\left( 1+\alpha e \right)e_{R}\geq0$.

It also can have non-trivial equilibrium $A_{b}\left( R_{A},P_{A} \right)$where

$R_{A}=\frac{\left( 1+e \right)e_{P}}{q},P_{A}(R)=\frac{q\left( R\left( K-R \right)-\left( 1+\alpha e \right)e_{R}K \right)}{R+b\left( 1+\alpha e \right)Kq}=\frac{Kq\left( 1+e \right)e_{P}-\left( 1+\alpha e \right)e_{R}Kq^{2}-\left( 1+e \right)e_{P}q^{2}}{\left( 1+e \right)e_{P}+b\left( 1+\alpha e \right)Kq^{2}}$ (A4.2)

In Fig. 7 we present the graph of null-isoclines of system (A4.1) and comparing it with those of system (A3.3) with $b=0$. We see that the graph of null-isocline $P_{A}\left( R \right), b>$0 is placed lower than $P_{A}\left( R \right), b=0$.

Stability and structures of equilibria $O,O_{1},O_{2}$, evidently, do not depend on parameter $b.$It is straightforward to verify that the determinant of the Jacobian matrix around equilibrium $A_{b}$ is the same as for the case $b=0$ and is given by expression (A3.19). So, as it was proven in Proposition 6, the point $A_{b}$ is non-saddle in domain of its positivity.

Trace of the Jacobian around $A_{b}$ is

$Tr\left( a \right)\equiv Tr(J_{V}\left( A_{b} \right)=\frac{\left( 1+e \right)e_{P}\boldsymbol{(}(-{(1+e)}^{2}{e_{P}}^{2}+(1+\alpha e)e_{R}Kq^{2})-b\boldsymbol{(}\boldsymbol{1+}\boldsymbol{\alpha}\boldsymbol{e}\boldsymbol{)K}\boldsymbol{q}^{\boldsymbol{2}}\boldsymbol{(2(1+e)}\boldsymbol{e}_{\boldsymbol{P}}\boldsymbol{+q)})}{(1+\alpha e)Kq^{2}((1+e)e_{P}+b\boldsymbol{(}\boldsymbol{1+}\boldsymbol{\alpha}\boldsymbol{e}\boldsymbol{)K}\boldsymbol{q}^{\boldsymbol{2}})}$ =

$$\left( e_{R}-\frac{\left( 1+e \right)^{2}{e_{P}}^{2}}{\left( 1+\alpha e \right)Kq^{2}} \right)-\left( \left( 1+e \right)e_{P}+Kq+\frac{\left( 1+\alpha e \right)e_{R}Kq^{2}}{\left( 1+e \right)e_{P}} \right)b+o\left( b^{2} \right)$$

=$Tr(J_{V}\left( A_{0} \right)-\left( \left( 1+e \right)e_{P}+Kq+\frac{\left( 1+\alpha e \right)e_{R}Kq^{2}}{\left( 1+e \right)e_{P}} \right)b+o(b^{2})$ (A4.3)

where

$\left( e_{R}-\frac{\left( 1+e \right)^{2}{e_{P}}^{2}}{\left( 1+\alpha e \right)Kq^{2}} \right)=Tr(J_{V}\left( A_{0} \right)$.

According to Appendix 3 $Tr(J_{V}\left( A_{0} \right)$=0 for $q=\frac{(1+e)\mathrm{ep}}{\sqrt{(1+\mathrm{al}e)\mathrm{er}K}} {\equiv q}_{0}.$ Searching the solution of the equation $Tr(J_{V}\left( A_{b} \right)$=0 in the form ${q=q}_{0}+\varepsilon b,$ we found $\varepsilon=\frac{{(1+e)}^{2}{e_{P}}^{2}(1+2\sqrt{\left( 1+\alpha e \right))e_{R}K})}{2 {e_{R}}^{2}\left( 1+\alpha e \right)}$ .

The stable limit cycle of model (A.4.1) that exists in Domain 5, with increasing of the parameter *q* disappears at the boundary *L* where the cycle coincides with the heteroclinic cycle composed by separatrices of saddles $O_{1}, O_{2}$; the boundary *L* is the same as for model (A.3.1).

The boundary $B\boldsymbol{:}e=\frac{K-4e_{R}}{4\alpha e_{R}}$ (see Theorem 1) separating Domains 1,2 from Domain 3 is the same for all systems (A.2,1), (A.3.1), and (A4.1)$.$

Thus, the following statement holds:

***Proposition 7.*** *Bifurcation diagram of system (A4.1) does not depend on parameter b for small values of b.*

REFERENCES

1. Andronov, A., Leontovich, E., Gordon, I., Maier, A. Qualitative theory of second-order dynamic systems, John Wiley &Sons, N.-Y.-Toronto, 1973.
2. Blanchard P, Devaney R.L., Hall G.R. Differential Equations. Brooks/Cole, 2011
3. Bautin, N., & Leontovich, E. *Methods and Techniques for Qualitative Analysis of Dynamical Systems on the Plane*. Moscow: Nauka, 1976. (in Rus.)
4. Bazykin, A.D., *Nonlinear dynamics of interacting populations*. World Scientific, World scientific series on Nonlinear Science, Ser. A, v.11, 2000.
5. Bazykin, A.D., Berezovskaya F.S. “*Allee effect, low critical density of population and predator-prey system dynamics*”, Problems of ecological monitoring and modeling of ecosystem. L.: Gidrometeoizdat, 1995, 309-328 (In Russian).
6. Kuznetsov Y. *Elements of applied bifurcation theory*. New York: Springer, 1998.
7. Volterra, V. *Le¸cons sur la Th´eorie Math´ematique de la Lutte pour la Vie*, Gauthier-Villare, Paris (1931).
